# Breast Cancer Preoperative Staging: Does Contrast-Enhanced Magnetic Resonance Mammography Modify Surgery?

**DOI:** 10.4061/2011/757234

**Published:** 2011-07-03

**Authors:** Chiara Perono Biacchiardi, Davide Brizzi, Franco Genta, Eugenio Zanon, Marco Camanni, Francesco Deltetto

**Affiliations:** ^1^Ginteam, Mini-Invasive Gynaecological and Breast Surgery Unit, Evangelical Hospital, ASL TO1, Via Silvio Pellico 19, 10125 Torino, Italy; ^2^Breast Radiology Unit, Evangelical Hospital, ASL TO1, Via Silvio Pellico 19, 10125 Torino, Italy

## Abstract

Women with newly diagnosed breast cancer may have lesions undetected by conventional imaging. Recently contrast-enhanced magnetic resonance mammography (CE-MRM) showed higher sensitivity in breast lesions detection. The present analysis was aimed at evaluating the benefit of preoperative CE-MRM in the surgical planning. From 2005 to 2009, 525 consecutive women (25–75 years) with breast cancer, newly diagnosed by mammography, ultrasound, and needle-biopsy, underwent CE-MRM. The median invasive tumour size was 19 mm. In 144 patients, CE-MRM identified additional lesions. After secondlook, 119 patients underwent additional biopsy. CE-MRM altered surgery in 118 patients: 57 received double lumpectomy or wider excision (41 beneficial), 41 required mastectomy (40 beneficial), and 20 underwent contra lateral surgery (18 beneficial). The overall false-positive rate was 27.1% (39/144). CE-MRM contributed significantly to the management of breast cancer, suggesting more extensive disease in 144/525 (27.4%) patients and changing the surgical plan in 118/525 (22.5%) patients (99/525, 18.8% beneficial).

## 1. Introduction

The primary objective of any diagnostic imaging modality is to accurately define the presence, the type, and the extent of disease in order to optimize patient management decisions and best plan therapeutic and surgical interventions. In women with suspected breast cancer, the aim of diagnostic imaging is to detect and accurately diagnose malignant tumors and to facilitate the correct choice of therapy, being mastectomy or breast-conserving surgery (e.g., lumpectomy) with or without preoperative neoadjuvant chemotherapy. The choice between breast-conserving surgery and mastectomy depends on numerous factors including tumour size, location and grade, the ratio of tumour size to breast volume, multifocality or multicentricity of the tumour, and patient preference. Currently, conventional mammography and ultrasound (US) are standard imaging techniques for the detection and evaluation of breast disease [1]. In recent years, contrast-enhanced magnetic resonance mammography (CE-MRM) has emerged as the most sensitive imaging modality for the detection and diagnosis of breast lesions [2–5]. Numerous studies have confirmed the superior diagnostic performance of CE-MRM compared to conventional mammography and US [6–9]. Studies to evaluate the impact of CE-MRM on patient management decisions have similarly revealed its superiority compared to standard imaging [10–13].

The present analysis was aimed at further evaluating the impact of CE-MRM on surgical decision making compared with those taken solely on the basis of clinical examination, conventional mammography, and ultrasound. The potential impact of CE-MRM on surgical decision making was, thereafter, evaluated for each patient. The CE-MRM was considered to accurately suggest the appropriateness of breast conservation images clearly which demonstrated the respectability of the lesion and in which CE-MRM was the only imaging modality able to do so. CE-MRM was considered to accurately suggest the necessity of changing surgery planning when images clearly showed more extensive disease than otherwise suspected from conventional mammography or ultrasound. More extensive disease includes larger size of index cancer, additional foci of cancer in the same or in other breast quadrants, and contra lateral lesions. Our purpose was to verify the benefit of preoperative CE-MRM in the surgical planning in our institution.

## 2. Materials and Methods

This retrospective study includes consecutive patients identified from a prospective database from January, 1, 2005 to November, 30, 2009. A standardized protocol was implemented in the management of all new, biopsy-proven breast cancer starting in January 2005. 

The primary inclusion *criterium* was a preoperative CE-MRM in patients with histologically confirmed breast cancer. The study included women 25 to 75 years of age with a new primary breast cancer. 

Exclusion *criteria* were mammographic pattern of fatty breast tissue, pregnancy, claustrophobia, planned bilateral mastectomy, preoperative chemotherapy, and history of breast cancer. 

All patients underwent mammography and ultrasonography. The evaluation of images was performed in consensus by four observers with 10 years' experience, respectively, in interpretation of conventional mammography and breast ultrasound images. Conventional mammograms and sonograms were evaluated for tumor detection and size. 

Needle biopsy was performed in case of suspicious lesion, often with radiographic (US or mammographic) guidance by 14 gauge core needle biopsy (Bard). 

Pathological results of core biopsy were in line with UK and European guidelines [14, 15]. Categories are B1: normal tissue/unsatisfactory; B2: benign; B3: lesions of uncertain malignant potential; B4: suspicious of malignancy; B5: (malignant subclassified as ductal carcinoma in situ (DCIS) or invasive cancer) [14, 15]. 

If the biopsy specimen was positive for malignancy, the patient was referred to surgeons. 

A complete clinical examination was performed and a preliminary surgical plan was made. Then, CE-MRM at 1.5 T was performed in the eligible patients.

CE-MRM was performed on a 1.5 T magnet (Achieva 1.5 T Philips) using a bilateral breast surface coil with the patient in the prone position.

An axial 3D dynamic T1-weighted gradient-echo sequence and T2-weighted pulse sequence were employed with images acquired before contrast agent administration (precontrast-unenhanced images) and, at 0, 1.5, 3, 4.5, and 6 minutes after the administration of contrast agent (postcontrast-enhanced images). Postcontrast 3D T1-weighted gradient-echo dynamic images were acquired after the administration of 0.1 mmol/kg bodyweight of gadopentetate dimeglumine Gd-DTPA (Magnevist Bayer Schering Pharma) through an 18 gauge needle cannula positioned in an antecubital vein. Gadopentetate dimeglumine Gd-DTPA was administered using an automatic injector at a rate of 2 mL/sec and was followed by 10 mL of saline solution at the same rate.

The evaluation of images was performed in consensus by two observers with 13 and 8 years' specific experience, respectively, in CE-MRM interpretation (approximately 1500 MR breast images per year).

If CE-MRM revealed more extensive breast disease, other than the index cancer, the patients would return for a second-look examination with mammogram and/or US. More extensive disease included larger size of index cancer, additional foci of cancer in the same or in other breast quadrants, and contra lateral lesions. 

Second look was performed by the same radiologists who interpreted the CE-MRM images. If a lesion was confirmed as suspicious, a new radiographic guided needle biopsy was performed. CE-MRM-guided biopsy is not available in our institution.

Whether the patients refused to undergo a core biopsy, additional surgery was strongly suggested. If the lesion was not seen on second look, the patient was counselled to remove it if the image was suspicious on CE-MRM, or to have 6-month followup CE-MRM if the lesion was less concerning in opinion of the attending breast radiologist. 

If the pathologic findings of the CE-MRM-discovered lesions biopsy specimen were malignant or high-risk pathology (atypical ductal hyperplasia (ADH), lobular intraepithelial neoplasia (LIN), papillary lesions, radial scar/complex sclerosing lesions), the case was reassessed by the same team of surgeons. A decision was made about the possibility to change surgical planning. There were three change's categories: first, from lumpectomy to double lumpectomy or wider excision, if the new lesions were located in the same quadrant but were separated from the index cancer by at least 1.0 cm of normal-appearing tissue on CE-MRM (multifocal lesions), or if there was a single additional lesion in other quadrant than index cancer (bicentric disease), or if it was in the same quadrant and contiguous with the original cancer or rounding it, but extended at least 4.0 cm beyond the site of the primary lesion (larger size); second, from breast conservative surgery to mastectomy, if lesions discovered were multicentric (more lesions in different quadrants), or if patient was not candidate to conservative surgery (e.g., retroareolar, large cancer in little breast); third, contra lateral surgery, if the lesions identified were in contra lateral breast.

After surgery, all radiographic and pathologic results were examined. 

In patients with a change of surgery, we analyzed tumour size and the presence of additional foci on mammographic, US, CE-MRM, and histologic reports to determine if the change of treatment was or not appropriate. Appropriate changes of treatment were defined as those in which pathologic report correlates with CE-MRM findings, but not with mammography and US. Inappropriate changes of surgery were those in which CE-MRM predicted a larger lesion or other foci than mammography or US, but the histological results confirmed the original mammographic and ultrasonographic findings. 

We defined as “false positive” patients, both with positive MRI and negative core biopsy, than with positive MRI and negative pathological report after surgery.

The institutional multidisciplinary breast conference of the Evangelical Hospital of Turin approved the employ of breast CE-MRM in women with newly diagnosed breast cancer, and the procedure was scheduled in the routinely workup of these patients after mammogram and US. The institutional review board of the Evangelical Hospital of Turin did not require the approval of patients, nor their informed consent to review their records on database. 

One-year followup was at least required to detect by mammogram or CE-MRM previously undetected lesions. About surveillance, we are in line with NCCN practice guidelines of invasive breast cancer. Physical exam and interval history every 4–6 months for 5 years, then every 12 months. Mammogram and US every 12 months (also MRI in recommended cases) [16]. 

Statistical analysis was performed using the Statistics Package for Social Sciences, version 15.0 (SPSS Inc., Chicago, Ill). Categorical variables were evaluated with *χ* ^2^ analysis. Results were considered statistically significant when  *P* < .05.

## 3. Results

During the 5-year study period, 525 women were defined eligible to undergo bilateral breast CE-MRM, following inclusion criteria. 

The mean age was 51.9 (range 25–75 years). Diagnosis of breast cancer was made by mammography and ultrasounds as seen in Table 1. 

The median invasive tumour size at study entry was 19 mm (range 1–60 mm), based on mammography/ultrasounds. 

In 302/525 patients (57.5%), breast cancer was a palpable mass and in 223/525 women (42.5%) presented with radiographic findings. 

Lumpectomy, double lumpectomy, or wider excision was performed for 396/525 patients (75.4%); 129/525 women (24.6%) underwent mastectomy. 

In 67/525 patients (12.8%), the definitive diagnosis was ductal carcinoma in situ, whereas in 458/525 (87.2%) cases was invasive carcinoma (Table 2).

A total of 190/458 patients (41.5%) with invasive cancer had lymph node-positive disease, preoperative, or after sentinel node biopsy (Table 3).

**Figure 1:**
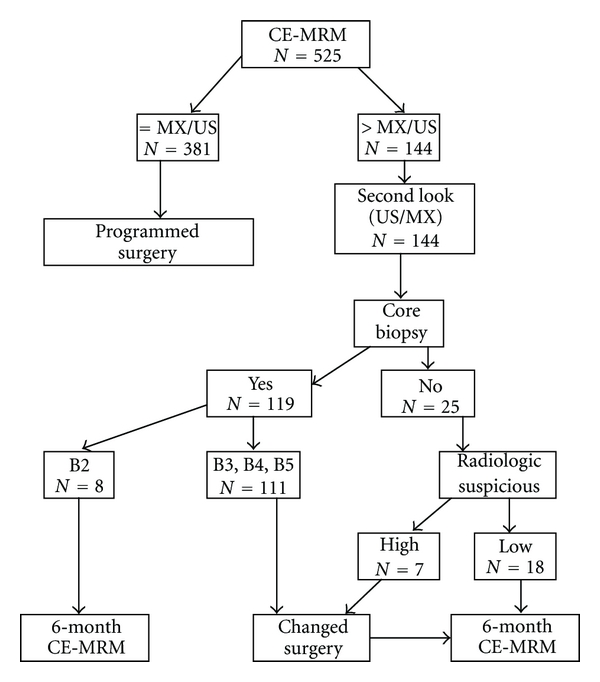
Additional evaluation based on breast CE-MRM findings and change in preoperative management. = MX/US: CE-MRM report in concordance with MX/US. > MX/US: CE-MRM detects more or larger lesions. B2: benign lesion; B3: lesion of uncertain malignant potential; B4: suspiciousnes of malignancy; B5: malignant (B5a: in situ carcinoma (DCIS) or B5b: invasive carcinoma) [14, 15]. *N*: number of patients.

At all, 525 women with a newly diagnosed breast cancer underwent CE-MRM according to the study protocol (Figure 1). CE-MRM findings were in concordance with mammogram and/or US in 381/525 patients (72.6%). 

In 144/525 patients (27.4%), CE-MRM identified suspicious lesions (Figure 1). In 26 patients, CE-MRM found additional images that resulted less concerning at second look with mammogram and/or US (18 cases) and benign at core biopsy (8 cases). In these cases, preoperative management unchanged and patients had six-month followup CE-MRM recommended. 

In 118 patients, CE-MRM detected lesions that the second look confirmed as concerning. A total of 111 patients underwent image-guided biopsy (US- or stereotactic-guided) which found B3, B4, or malignancy in the specimens [14, 15]. In 7 patients (4 patients who refused to have a new core biopsy and 3 patients in which the second look did not identify the additional enhancing lesion detected by CE-MRM), on the basis of high suspect of CE-MRM imaging, patients were strongly recommended to undergo to wider surgery (Figure 1). 

CE-MRM altered programmed surgery of newly diagnosed breast cancers in 118/525 (22.5%) patients (Table 4). Fifty-seven patients who were initially candidates for breast-conserving surgery were upgraded, based on CE-MRM findings, to double lumpectomy or to wider excision. In 20/57 patients, CE-MRM found additional foci, and in 37/57 patients, the size of index cancer was larger. 

On the basis of CE-MRM imaging, 41 women required a mastectomy. 37/41 patients had multicentric cancer CE-MRM detected, in 4/41 patients, there were a larger lesion with unfavourable cancer size/breast size ratio. 

All patients enrolled in the study received bilateral CE-MRM, and 20 women had suspicious lesions discovered in the contra lateral breast (Table 4). 

Of these 20 patients, all demonstrated with needle biopsy, 6 women had programmed operation in the ipsilateral breast and a new contra lateral surgery; in 14 patients, the surgical plan changed bilaterally, according to the additional lesions detected by CE-MRM.

A radiographic-pathologic correlation was performed to verify whether the change in surgical management based on CE-MRM was beneficial, owing to better concordance between CE-MRM and surgical pathologic findings than between mammography or US and histological reports. 

CE-MRM detected enhanced lesions in 144 cases (Figure 1). The second look identified suspicious lesions in 126 cases, and, in 119 patients, an image-guided biopsy (ultrasonographic or stereotactic) was performed. Pathologic reports confirmed an apparent malignancy in the specimens in 111 patients, whereas 8 patients had benign lesions. The false-positive rate for biopsy of a CE-MRM-detected lesion was 8/119 (6.7%). In 18 patients who refused to undergo core-biopsy after second look, the lesions were considered by our radiologist as less concerning; these patients had six-month CE-MRM followup recommended. Therefore, the total false-positive rate for second look was 26/144 (18%).

As illustrated in Figure 1, 118 patients had a change in surgical plan. In 13 patients, change of surgery was inappropriate (Table 4), in 11 patients, in which wider excision was performed, histological reports did not confirm CE-MRM suggestions, lesions were smaller than 4.0 cm, or the second lesion identified was near the index cancer (distance < 1.0 cm). In one patients in which wider excision and contra lateral surgery were performed, histology demonstrated that surgery was appropriate in the breast with index cancer, but, in contra lateral breast, definitive diagnosis was benign. Finally, in one patient who had >4.0 cm CE-MRM-detected lesion, operation was converted to mastectomy, but the surgical histological report did not confirm CE-MRM findings. The false-positive rate for surgery was 13/118 (11%). 

In summary, the overall false positive rate was 39/144 (27.1%).

As seen in Table 4, in six-women breast, CE-MRM detected additional separate lesions (4 patients), or it confirmed the presence of the known lesion, but larger (2 patients), which allowed a wider excision (Table 4). Unfortunately, histology demonstrated the presence of more extensive disease (6/118, 5% false-negative rate). 

Therefore, among 118 patients who had a change in surgical plan, 99 (84%) were found to have a concordance between CE-MRM findings and final histological reports. Surgical change was defined in these patients appropriate and beneficial (Table 4). Forty one of the 57 women (71.9%) who had an initially planned lumpectomy converted to a double lumpectomy or to a wider excision based on CE-MRM were converted appropriately. Forty of 41 patients (97.6%) who had a lumpectomy converted to a mastectomy had a beneficial change because CE-MRM correlated with final pathologic report. In the 20 women with contra lateral CE-MRM-detected lesions, the histological report correlated with CE-MRM findings in 19 (95%).

In 163/525 patients, breast cancer was multicentric (31%). In 88/163 patients, breast cancer was defined as multicentric before CE-MRM. In seventy-five patients of 163 (46%), we modified surgical planning because CE-MRM detected additional foci of breast cancer (including also bicentric disease, in which double lumpectomy was performed). 

On univariate analysis, we considered patient age, radiographic findings, pathologic features, and staging. We considered patients divided into the three types of changed surgery. We focused our attention on interesting results (see Tables 5-7). 

We found that patients with ILC (7/64, 9.5%) were more likely to have contra lateral disease compared with IDC (6/287, 2.1%); *P* < .0001 (Table 5). Patients with positive nodes (25/180, 13.9%) were converted to mastectomy more often than women with negative nodes (16/325, 4.9%); *P* < .0001 (Table 6). Similarly, we found that patients with multicentric disease were more likely to have mastectomy (37/145, 25.5% versus 4/360, 1.1%; *P* < .0001) and contra lateral breast cancer (18/163, 11.0% versus 2/362, 0.5%; *P* < .0001), compared with patients with unifocal breast cancer (Table 7). 

**Figure 2:**
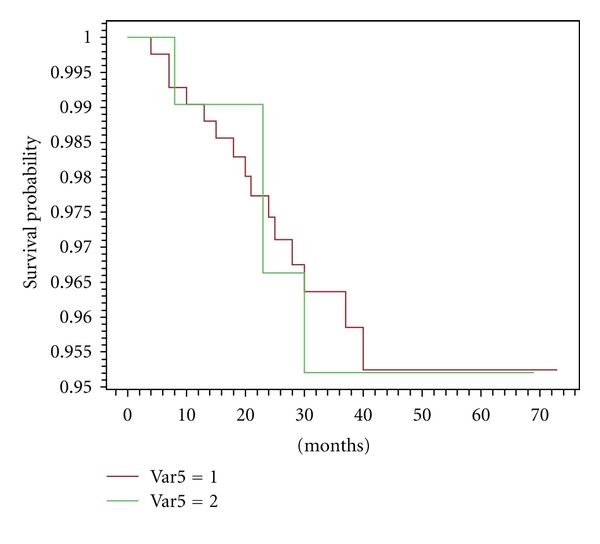
Curves of disease-free survival local recurrences.Var5 1: patients with unmodified surgery after CE-MRM Var5 2: patients with modified surgery after CE-MRM *P* = .97.

**Figure 3:**
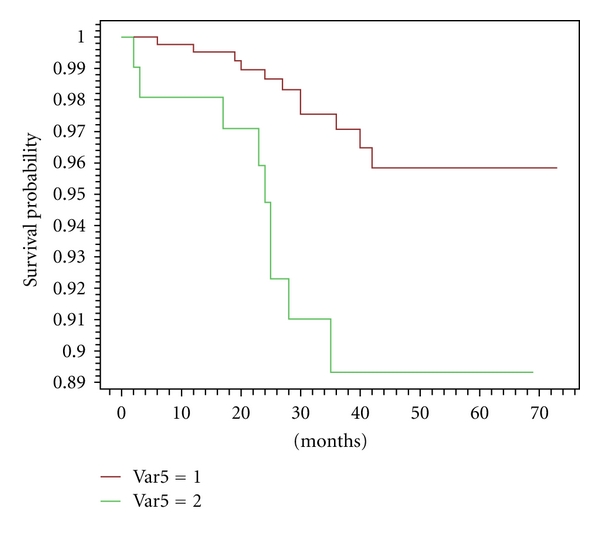
Curves of disease-free survival distant metastases. Var5 1: patients with unmodified surgery after CE-MRM Var5 2: patients with modified surgery after CE-MRM *P* = .002.

**Figure 4:**
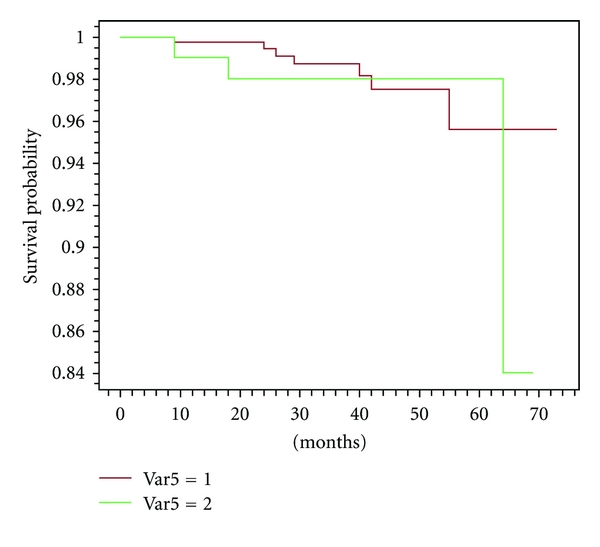
Curves of overall survival.

The number and the site of recurrences are reported in Table 8. In our series, as expected and hoped, the number of first local failures was similar in women with converted surgery, compared with patients with any change of treatment (Figure 2); however, we notice that the number of distant metastases seems to be higher in cases with modified surgery versus unmodified surgery. Kaplan-Meier survival analysis (distant disease-free and overall survival) showed both curves overlapping around 97% at 5 years (Figures 3 and 4). Considering the short followup (median 36 months), firm statistical conclusions are hard. 

## 4. Discussion

This retrospective study evaluated the impact of CE-MRM on the surgical management of 525 consecutive patients of 25–75 years of age with newly diagnosed breast cancer. 

Since CE-MRM is performed in all the patients in our hospital (except patients >75-year old and patients with mammographic pattern of fatty breast tissue), only few patients were left out of the study. 

Patients were treated following a workup in which our breast surgeons assessed all patients before CE-MRM. Women were all revaluated after CE-MRM by the same surgeons to decide if a change in surgical planning was necessary. 

CE-MRM-altered programmed surgery in 118/525 (22.5%) of patients and, based on findings founded in the pathologic specimens, the change of surgery planning was confirmed as appropriate in 99/118 (84%) of these patients. Thus, 99/525 (18.8%) of women had a favourable change in surgical management, based on preoperative CE-MRM. Therefore, 5 women must undergo to CE-MRM for 1 to have a beneficial conversion in surgical plan.

Surgical management, other than the histology and the size of the breast, is usually influenced by the real size of index cancer and by the extent of the disease, indicated by the presence of multiple malignant foci in the same quadrant or in different quadrants from the main lesions, or by the presence of contra lateral lesions. CE-MRM has demonstrated that, despite its suboptimal specificity, it is able to offer this kind of information better than conventional radiology. 

The first risk of CE-MRM is, in fact, the number of false-positive that may cause unnecessary imaging and biopsies, and that is a major limitation in the use of this procedure [17]. In this regard, false positives (and also false negatives) after CE-MRM can be attributed to inherent technological limitation of CE-MRM, patients characteristics, quality assurance failures, and human error [18]. The consequences of these factors include missed cancers, with potentially worse prognosis, as well as anxiety and potential harms associated with interventions for benign lesions [18]. 

In our series, the overall false-positive rate was 39/144 (27.1%), in which CE-MRM-detected lesions were ultimately not malignant. In 13/118 (11%) of patients in which change of surgery was decided (13/118), the conversion was inappropriate (Table 4). 

Furthermore, 11 women were upgraded from lumpectomy to wider excision or double lumpectomy, but histological reports did not confirm CE-MRM suggestions. Analyzing these records in our database, we verified that four patients refused to have a guided core biopsy after second look; two patients had a negative second look with high suspiciousess of CE-MRM findings. In these cases, the pathologic specimens revealed the presence of benign lesions. The other cases were B3 and B4 as result of core biopsy. Definitive pathologic reports verified that lesions resulted are not malignant. 

In one patient in which ipsilateral wider excision and contra lateral surgery were performed, histology demonstrated that surgery was appropriate in the breast with index cancer, but, in contra lateral breast final diagnosis was LIN 1. 

Moreover, in one patient, an unnecessary mastectomy was programmed, because the lesion was overestimated by CE-MRM, and, in the histological specimen, the presence of a LIN 1 near the index cancer was verified. 

Considering false negatives, in six of 118 women, CE-MRM detected additional lesions, which allowed a wider excision. These patients were borderline candidates for breast-conserving therapy, and, after an exhaustive counselling with them, the decision to attempt a wider excision was made. Unfortunately, histology demonstrated the presence of more extensive disease (5% false-negative rate), and a subsequent mastectomy was performed. 

Numerous reports showed that CE-MRM can detect additional foci in a substantial number of women with a new diagnosis of breast cancer [6–9]. Moreover, numerous nonrandomized studies have attempted to evaluate the effect of CE-MRM on surgical treatment and planning [10–13]. The only evidence from a prospective randomized trial on the impact of CE-MRM on surgical management derived from the COMICE study [19], a controlled randomized trial that was designed to measure the reexcision rate as its primary endpoint (Turnbull et al., 2010). In this trial 1,625 women were randomly evaluated before surgery with breast CE-MRM or not [19]. Reexcision rates were quite similar in women randomized to receive conventional assessment (19.3%) or to receive CE-MRM in addition to standard imaging (18.8%); NS [19].

Previous reports have also described the identification of previously undetected, synchronous lesions in the contra lateral breast using CE-MRM in an average of 5% of women with a recent diagnosis of breast cancer [20–22]. 

The most of CE-MRM-detected contra lateral breast cancers appear to early stage disease, as indicated in a recent review [23], and, in approximately 2/3 of cases, the specimens were positive for invasive cancer [23, 24].

In patients with invasive lobular carcinoma (ILC), the coexistence of other invasive malignant foci, identified by breast CE-MRM, apart from the index lesion in the ipsilateral breast reached 32% in a recent meta-analysis [25]. Moreover, the detection rate of contra lateral ILC is another 7% of patients by CE-MRM only [25].

Our overall detection rate of contra lateral breast cancer was 20/525 (3.8%). All the contra lateral lesions CE-MRM detected were guided-biopsy proven and only one of them were overestimated. CE-MRM (Table 5) identified bilaterality in 3/67 (4.5%) of DCIS in 6/287 (2.1%) of IDC and in 7/74 (9.5%; *P* < .0001) of ILC, respectively. Finally, the number of the CE-MRM-detected contra lateral breast cancers was unrelated to nodal status (Table 6). The fact that change in treatment was considered correct, as verified by pathologic findings in the specimen, in 19/20 (95%) of cases of contra lateral surgery (Table 4), shows that breast cancer, and especially ILC, is often more extensive than appreciate on conventional imaging. 

Our study shows that the CE-MRM can improve the detection of other malignant lesions (ipsilateral and contra lateral) when added to a conventional imaging (mammogram and US) at the time of the initial diagnosis of breast cancer. The current cost of CE-MRM precludes its widespread use in general population, but this imaging tool appears to improve the detection of cancer in women at increased risk, such as women with a recent diagnosis of breast cancer, and a number needed to treat of 5 is reasonable in our opinion.

If CE-MRM is performed, the false-positive rate indicates that abnormal findings should be investigated with image-guided core biopsy to establish a diagnosis before surgical treatment, as emphasized in a recent review [26].

The second risk of this approach to local staging the breast is that more women being treated with more radical surgery without a demonstrated improvement in surgical outcomes or prognosis.

Based on the results of controlled clinical trials with mortality as the endpoint, breast conservation therapy (BCT) and mastectomy confer equivalent risk to the patient [27–29]. As stated by Orel and Schnall [4], the 25–36% of local recurrence rate in the absence of radiotherapy and chemotherapy corresponds to the frequency of multifocal and multicentric tumours found only with conventional imaging [4]. The potential 10-year recurrence rate after breast-conserving therapy followed by standard adjuvant therapies (radiation therapy, chemotherapy, and hormonal therapy) would be 9-10% [30]. Moreover, the absolute risk of contra lateral breast cancer in women with a personal history of breast cancer is up to 3% of synchronous disease, whereas 7% of women will be diagnosed with metachronous disease [22]. This risk is significantly higher than that of the general population [31]. In this regard, adjuvant therapies (local and systemic) play a key role in achieving local control in women treated with breast-conserving surgery. Thus, the goal of breast-conserving therapy is to achieve good local control, and to provide women who wish to conserve their breast a good cosmetic result. 

Some argue that any increase in the rate of mastectomy prompted by CE-MRM findings would represent a setback in the standard of care [32, 33]. And since radiation therapy is presumed to eradicate or delay the progression of residual disease in most women who undergo conservation therapy, preoperative CE-MRM would have little or no impact on rates of recurrence or death [32]. 

On the other hand, the upper threshold amount of residual disease that can be eradicated by radiation therapy is not yet well established. Although the rate of recurrence after breast conservation is low, it is not zero, and each patient should be offered the best possible chance for successful treatment. Detecting widespread disease can obviate inappropriate attempts at conservation, in which both lumpectomy with positive margins and reexcision with positive margins are carried out before the full extent of the disease burden is understood. A staging CE-MRM examination showing only a single cancer lesion may permit the patient to choose conservation therapy with a degree of confidence that no macroscopic disease will be missed at surgery [34]. About our false positives, as yet explained above, the pathologic reports described four cases of ADH, and two cases of LIN 1. In women with ADH a review of literature suggests a 4- to 5-fold increased risk of invasive breast cancer, compared with a 6- to 10-fold risk ALH/LIN [35]. With regard to lobular neoplasia, the subsequent invasive cancer may be ipsilateral or contra lateral, and more than 50% of these diagnoses occur more than 15 years after the original diagnosis of lobular neoplasia [36, 37]. Thus, in our opinion, the excision of these lesions that were considered clinical risk factors of breast cancer was absolutely correct. 

Therefore, if we believe that it is important to clear lumpectomy margins of breast disease (from atypical hyperplasia to in situ and microinvasive carcinoma) to reduce the risk of local recurrence, it should follow that small foci in both breast detected on CE-MRM also warrant identification and excision.

After a median followup of 36 months, we reported 5/118 (4.2%) versus 15/407 (3.7%) (NS) local recurrences in women with converted surgery, compared with patients with any change of treatment (Figure 2). However, the two populations differed as regard the metastatic risk, so much as to be able to undo the effect of possible local benefit. In our series, we observed higher rates of larger cancers > pT1 (39/118, 33.0% versus 92/407, 22.6%) and of nodal involvement (58/118, 49.1 versus 144/407, 35.4; *P* < .0001) in cases with modified versus unmodified surgery. This condition could carry out the higher rate of distant recurrences. In fact, we observed up to this time 9/118 (7.6%) events in former group versus 11/407 (2.7%) in the latter. These few events do not allow us to distinguish any subgroup of risk, that is multicentricity versus larger size of tumour. Kaplan-Meier survival analysis showed both curves overlapping around 97% at 5 years (Figures 3-4). As reported elsewhere [12], larger tumour size is an independent factor of a beneficial change in surgical management of newly diagnosed breast cancer in patients who undergo CE-MRM (odds ratio 1.66; 95% C.I., 1.04–2.66) [12]. 

Anyway, before to say that CE-MRM have little or no impact on local recurrence rate and on survival rate, because women are at higher risk of distant metastases, the number of observations and the followup should be implemented.

## 5. Conclusions

The use of CE-MRM results in a beneficial change in surgical management in 99/525 (18.8%) of patients. Additional malignant lesions are detected in about one patient every five who undergo CE-MRM. 

These data suggest that CE-MRM plays a role in the staging evaluation of newly diagnosed breast cancers.

Our experience confirms that, when needle-biopsy was missed, the suspiciousness of CE-MRM-imaging findings was not sufficient to advise a change in surgical planning (six high suspicious CE-MRM-detected lesions without preoperative histological confirmation resulted benign after surgery). Thus, we conclude that guided needle biopsy is always recommended to verify additional CE-MRM-detected lesions. 

This study has some limitation to be addressed. It is a retrospective report of consecutive patients with a proven diagnosis of newly breast cancer. The number of patients is quite large, but the median followup is not so long to make firm statistical conclusions.

Therefore, future research is mandatory to explore the value of CE-MRM in the improvement of surgical outcome and prognosis by decreasing the need for reoperation and lowering recurrent rates.

## Figures and Tables

**Table 1:** Breast cancer diagnosis.

	*N (%)*
Positive MX + positive US	401 (76.4)
Positive MX + negative US	70 (13.3)
Negative MX + positive US	54 (10.3)

Total	525 (100)

MX: mammography.

US: ultrasounds.

*N*: number of patients.

**Table 2:** Histopathologic types.

	*N (%)*
DCIS	67 (12.8)
Invasive carcinomas	458 (87.2)
(i) ductal (ii) lobular (iii) others	287 (63) 74 (16) 97 (21)

Total	525 (100)

DCIS: ductal carcinoma in situ.

*N*: number of patients.

Others: ductal-lobular (49); mucinous (15); tubular (14); medullary (9); metaplastic (3); papillary (7).

**Table 3:** Axillary nodes status.

Evaluation	*N* (%)
Positive nodes: (i) FNAC + (ii) SNB +	91 (19.9) 99 (21.6)
Negative SNB	268 (58.5)

Total	458 (100)

SNB: sentinel node biopsy.

FNAC: fine needle aspiration cytology.

*N*: number of patients.

**Table 4:** Change in surgical management based on CE-MRM.

Treatment change	Change	Beneficial	FP	FN
	*N* (%)	*N* (%)	*N* (%)	*N* (%)
(A) Double lumpectomy or wider excision	57 (48.3)	41 (71.9)	11 (19.3)	5 (8.8)
(B) Mastectomy	41 (34.7)	40 (97.6)	1 (2.4)	0
(C) Contra lateral surgery: (i) alone (ii) in addition to (A) (iii) in addition to (B)	6 (5.1) 8 (6.8) 6 (5.1)	6 (100) 6 (75) 6 (100)	0 1 (12.5) 0	0 1 (12.5) 0

Total	118 (100)	99 (84)	13 (11)	6 (5)

*N*: number of patients.

FP: false positives.

FN: false negatives.

**Table 5:** Histopatologic type in the subgroups.

	Patients	Double lumpectomy/Wider excision*	Mastectomy*	Contra lateral surgery
	*N *	*N* (%)	*N* (%)	*N* (%)
DCIS	67	6 (9)	5 (7.5)	3 (4.5)
IDC	287	31 (10.8)	25 (8.7)	6 (2.1)
ILC	74	10 (13.5)	7 (9.5)	7 (9.5)**
Others	97	10 (10.3)	4 (4.1)	4 (4.1)

DCIS: ductal carcinoma in situ; IDC: infiltrating ductal carcinoma; ILC: infiltrating lobular carcinoma.

*Patients with synchronous contra lateral surgery were excluded.

**Lobular versus ductal histotype *P* < .011.

Others: ductal lobular (49); mucinous (15); tubular (14); medullary (9); metaplastic (3); papillary (7).

**Table 6:** Negative versus positive nodes in the subgroups.

	Double lumpectomy/Wider excision*	Mastectomy*	Contra lateral surgery
Negative nodes (%) Positive nodes (%) *P* value	32/325 (9.8) 25/180 (13.9) (NS)	16/325 (4.9) 25/180 (13.9) (*P*<.0001)	10/335 (3.0) 10/190 (5.3) (NS)

*Patients with synchronous contra lateral surgery were excluded.

**Table 7:** Multicentric versus unifocal cancer in the subgroups.

	Double lumpectomy/Wider excision*	Mastectomy*	Contra lateral surgery
Multicentric (%) Unifocal (%) *P* value	20/145 (13.8) 37/360 (10.3) (NS)	37/145 (25.5) 4/360 (1.1) (*P* < .0001)	18/163 (11.0) 2/362 (0.5) (*P* < .0001)

*Patients with synchronous contra lateral surgery were excluded.

**Table 8:** Site and number of first failure after treatment.

	Change surgery	
	No	Yes
Site	*N* (%)	*N* (%)
Local	15*(3.7)	5**(4.2)
Regional	3 (0.7)	1 (0.8)
Contra lateral breast cancer	5***(1.2)	0
Distant	11 (2.7)	9 (7.6)
None	373 (91.6)	103 (87.3)

*Includes one patient with concurrent contra lateral breast cancer and four patients with synchronous distant metastases.

**Includes one patient with concurrent distant metastases.

***Includes one patients with concurrent ipsilateral local failure.

## References

[1] Smith RA, Saslow D, Andrews Sawyer K (2003). American Cancer Society Guidelines for breast cancer screening: update 2003. *Ca-A Cancer Journal for Clinicians*.

[2] Heywang-Köbrunner SH, Bick U, Bradley WG (2001). International investigation of breast MRI: results of a multicentre study (11 sites) concerning diagnostic parameters for contrast-enhanced MRI based on 519 histopathologically correlated lesions. *European Radiology*.

[3] Sardanelli F, Giuseppetti GM, Panizza P (2004). Sensitivity of MRI versus mammography for detecting foci of multifocal, multicentric breast cancer in fatty and dense breasts using the whole-breast pathologic examination as a gold standard. *American Journal of Roentgenology*.

[4] Orel SG, Schnall MD (2001). MR imaging of the breast for the detection, diagnosis, and staging of breast cancer. *Radiology*.

[5] Kuhl C (2007). The current status of breast MR imaging. Part I. Choice of technique, image interpretation, diagnostic accuracy, and transfer to clinical practice. *Radiology*.

[6] Boetes C, Mus RDM, Holland R (1995). Breast tumors: comparative accuracy of MR imaging relative to mammography and US for demonstrating extent. *Radiology*.

[7] Lee JM, Orel SG, Czerniecki BJ, Solin LJ, Schnall MD (2004). MRI before reexcision surgery in patients with breast cancer. *American Journal of Roentgenology*.

[8] Kriege M, Brekelmans CTM, Boetes C (2004). Efficacy of MRI and mammography for breast-cancer screening in women with a familial or genetic predisposition. *The New England Journal of Medicine*.

[9] Kuhl CK, Schrading S, Leutner CC (2005). Mammography, breast ultrasound, and magnetic resonance imaging for surveillance of women at high familial risk for breast cancer. *Journal of Clinical Oncology*.

[10] Fischer U, Kopka L, Grabbe E (1999). Breast carcinoma: effect of preoperative contrast-enhanced MR imaging on the therapeutic approach. *Radiology*.

[11] Del Frate C, Borghese L, Cedolini C (2007). Role of pre-surgical breast MRI in the management of invasive breast carcinoma. *Breast*.

[12] Bilimoria KY, Cambic A, Hansen NM, Bethke KP (2007). Evaluating the impact of preoperative breast magnetic resonance imaging on the surgical management of newly diagnosed breast cancers. *Archives of Surgery*.

[13] Berg WA, Gutierrez L, NessAiver MS (2004). Diagnostic accuracy of mammography, clinical examination, US, and MR imaging in preoperative assessment of breast cancer. *Radiology*.

[14] NHS Non-Operative Diagnosis Subgroup of the National Coordination Group for Breast Screening Pathology Guidelines for Non-operative Diagnostic Proededures and Reporting in Breast Cancer Screening.

[15] Perry N, Broeders M, De Wolf C (2006). *European Guidelines for Quality Assurance in Breast Cancer Screening and Diagnosis*.

[16] National Comprehensive Cancer Network (2010). *Clinical Practice Guideline in Oncology—Breast Cancer, Version 2*.

[17] Houssami N, Hayes DF (2009). Review of preoperative magnetic resonance imaging (MRI) in breast cancer: should MRI be performed on all women with newly diagnosed, early stage breast cancer?. *CA Cancer Journal for Clinicians*.

[18] Saslow D, Boetes C, Burke W (2007). American Cancer Society guidelines for breast screening with MRI as an adjunct to mammography. *CA Cancer Journal for Clinicians*.

[19] Turnbull LW, Brown SR, Olivier C (2010). Multicentre randomised controlled trial examining the cost-effectiveness of contrast-enhanced high field magnetic resonance imaging in women with primary breast cancer scheduled for wide local excision (COMICE). *Health Technology Assessment*.

[20] Liberman L, Morris EA, Kim CM (2003). MR Imaging findings in the contralateral breast of women with recently diagnosed breast cancer. *American Journal of Roentgenology*.

[21] Kuhl C, Schmiedel A, Morakkabiti N (2000). Breast MR imaging of the asymptomatic contralateral breast in the work-up or follow-up of patients with unilateral breast cancer. *Radiology*.

[22] Lee SG, Orel SG, Woo IJ (2003). MR imaging screening of the contralateral breast in patients with newly diagnosed breast cancer: preliminary results. *Radiology*.

[23] Brennan ME, Houssami N, Lord S (2009). Magnetic resonance imaging screening of the contralateral breast in women with newly diagnosed breast cancer: systematic review and meta-analysis of incremental cancer detection and impact on surgical management. *Journal of Clinical Oncology*.

[24] Lehman CD, Gatsonis C, Kuhl CK (2007). MRI evaluation of the contralateral breast in women with recently diagnosed breast cancer. *The New England Journal of Medicine*.

[25] Mann RM, Hoogeveen YL, Blickman JG, Boetes C (2008). MRI compared to conventional diagnostic work-up in the detection and evaluation of invasive lobular carcinoma of the breast: a review of existing literature. *Breast Cancer Research and Treatment*.

[26] Lehman CD, DeMartini W, Anderson BO, Edge SB (2009). Indications for breast MRI in the patient with newly diagnosed breast cancer. *Journal of the National Comprehensive Cancer Network*.

[27] Fisher B, Anderson S, Redmond CK, Wolmark N, Wickerham DL, Cronin WM (1995). Reanalysis and results after 12 years of follow-up in a randomized clinical trial comparing total mastectomy with lumpectomy with or without irradiation in the treatment of breast cancer. *The New England Journal of Medicine*.

[28] Veronesi U, Cascinelli N, Mariani L (2002). Twenty-year follow-up of a randomized study comparing breast-conserving surgery with radical mastectomy for early breast cancer. *The New England Journal of Medicine*.

[29] Morris AD, Morris RD, Wilson JF (1997). Breast-conserving therapy vs mastectomy in early-stage breast cancer: a meta-analysis of 10-year survival. *Cancer Journal from Scientific American*.

[30] Clarke M, Collins R, Darby S (2005). Effects of radiotherapy and of differences in the extent of surgery for early breast cancer on local recurrence and 15-year survival: an overview of the randomised trials. *The Lancet*.

[31] Fowble B, Hanlon A, Freedman G, Nicolaou N, Anderson P (2001). Second cancers after conservative surgery and radiation for stages I-II breast cancer: identifying a subset of women at increased risk. *International Journal of Radiation Oncology Biology Physics*.

[32] Morrow M, Freedman G (2006). A clinical oncology perspective on the use of breast MR. *Magnetic Resonance Imaging Clinics of North America*.

[33] Kuhl C, Kuhn W, Braun M, Schild H (2007). Pre-operative staging of breast cancer with breast MRI: one step forward, two steps back?. *Breast*.

[34] Schnall M (2006). MR imaging evaluation of cancer extent: is there clinical relevance?. *Magnetic Resonance Imaging Clinics of North America*.

[35] Arpino G, Laucirica R, Elledge RM (2005). Premalignant and in situ breast disease: biology and clinical implications. *Annals of Internal Medicine*.

[36] Fisher ER, Land SR, Fisher B, Mamounas E, Gilarski L, Wolmark N (2004). Pathologic findings from the national surgical adjuvant breast and bowel project: twelve-year observations concerning lobular carcinoma in situ. *Cancer*.

[37] Li CI, Malone KE, Saltzman BS, Daling JR (2006). Risk of invasive breast carcinoma among women diagnosed with ductal carcinoma in situ and lobular carcinoma in situ, 1988–2001. *Cancer*.

